# Genetics in the 21st Century: Implications for patients, consumers and citizens

**DOI:** 10.12688/f1000research.12850.2

**Published:** 2018-01-24

**Authors:** Jonathan Roberts, Anna Middleton

**Affiliations:** 1 Society and Ethics Research Group, Connecting Science, Cambridge, CB10 1SA, UK

**Keywords:** genomics, patient, citizen, consumer

## Abstract

The first human genome project, completed in 2003, uncovered the genetic building blocks of humankind. Painstakingly cataloguing the basic constituents of our DNA (‘genome sequencing’) took ten years, over three billion dollars and was a multinational collaboration. Since then, our ability to sequence genomes has been finessed so much that by 2018 it is possible to explore the 20,000 or so human genes for under £1000, in a matter of days. Such testing offers clues to our past, present and future health, as well as information about how we respond to medications so that truly ‘personalised medicine’ is now moving closer to a reality.

The impact of such a ‘genomic era’ is likely to have some level of impact on an increasingly large number of us, even if we are not directly using healthcare services ourselves. We explore how advancements in genetics are likely to be experienced by people, as patients, consumers and citizens; and urge policy makers to take stock of the pervasive nature of the technology as well as the human response to it.

## Introduction

Genomic technology is now being utilised in more settings across society than ever before, including medicine, population health screening, recreational consumerism (ancestry testing, nutritional testing), through to policing and crime prevention. Signifying the importance placed on genomics, the most recent annual report of the Chief Medical Officer of the United Kingdom (2017) was entitled “Generation Genome” and stated:

"Genomics is not tomorrow. It’s here today. I believe genomic services should be available to more patients, whilst being a cost-effective service in the NHS. This is exciting science with the potential for fantastic improvements in prevention, health protection and patient outcomes. Now we need to welcome the genomic era and deliver the genomic dream!" ^1^

As members of the public we are all likely to all be exposed to some level of genomics within our lifetime – whether it be directly via a medical test, indirectly through marketing for recreational testing or distantly through being biologically related to someone whose DNA is contained within a national database. Given that genomic information links us to our relatives, the decisions that we make about it (whether to donate it for research, whether to be tested, whether to withhold it) will all have an impact on those we are related to and the knowledge that they too can gain. It is this fact that makes genetic information quite different from other sorts of medical information. Thus, we all have a stake in policy that guides the utilisation of genomic data. Within this opinion article, we reflect upon some of the ways that genomic data may touch us as patients, consumers and citizens.

Before continuing it is important to clarify that in this article we use the term ‘genomic’ to refer to a wide range of sequencing and laboratory techniques. There are a range of different technologies that interrogate the ‘whole genome’. Next generation sequencing may be used to create a ‘whole genome sequence’ - a complete catalogue of all (approx) 3 billion base pairs of an individual’s genome. There are also a number of technologies that look across an entire genome but do not create a ‘whole genome sequence’ for example, microarrays, SNP arrays, and exome sequences. For our purposes we refer to all technologies that look across the genome as ‘genomic’. Where there are specific technological issues (such as positive and negative predictive values in direct to consumer genetic tests) we refer to these. For the purposes of this article we discuss broadly the implications of ‘genomic’ technology with an understanding that this refers to a range of sequencing and testing techniques. We also use the term ‘genetics’ broadly with an understanding that this term covers wide disciplinary boundaries, including genomics.

### People as patients

The UK has been an early implementer of genomics in a healthcare setting. In 2014, the Deciphering Developmental Disorders Study reported the first results from the sequencing of 12,000 children with developmental disorders ^2^. At the same time, work began on a project to sequence 100,000 genomes of 70,000 NHS patients ^3^. Outside of the UK, sequencing in healthcare settings is also expanding. For example, the National Institutes of Health Precision Medicine Initiative in the US offers 1 million people some level of genomic sequencing ^4^ and the Australian Genomics Health Alliance is currently creating the infrastructure to integrate genomic medicine into healthcare nationally. Possibly the most striking example comes form Qatar where the government has plans to offer genome sequencing to their entire population ( www.qatargenome.org.qa/).

A commitment to significant and ongoing investment in genomics on an international scale is demonstrated by the ‘Million European Genomes Project’, where genomic and health data will be linked from 1 million European patients ^5^ and The Global Alliance for Genomics and Health ( www.ga4gh.org) that represents more than 500+ organisations globally all with the shared endeavour of implementing and enabling genomic data sharing to be applied in research and clinical practice.

Genomic technology is now being utilised globally in a wide range of contexts to provide diagnostic, prognostic and treatment information for patients in a way that is predicted to transform healthcare ^1^. Genetic testing is currently used in a range of hospital settings, including the diagnosis of rare disease in paediatrics, prenatal care, ophthalmology, dermatology, ENT, etc. Such testing will continue to be used in these settings. However, as genetic testing (single genes) shifts to genomic testing (many genes) and the ability to test becomes faster and cheaper, a key change that will occur is a dramatic increase in the amount of information generated. As an example, as recently as 5 years ago, a breast cancer patient who also has family history of young onset breast cancer would likely be tested in a healthcare setting for two genes (BRCA1 and BRCA2). Alterations in these genes are known to lead an increased risk for certain types of cancer, most notably breast and ovarian cancer. Whereas now, such a patient may have a ‘panel test’ where over 20 genes associated with breast cancer are explored ^6^. Each of these genes will have different links to cancer, thus increasing the complexity of the results that emerge. In addition to using genomic technology to understand the genetic basis of an existing condition such as breast cancer, it can also be used to uncover a new diagnosis for a previously undescribed rare condition ^7^. Again, here the key change is the volume of information that will be generated. Instead of testing just a few genes, a child with an unknown developmental disorder may now have 20,000+ genes sequenced and then filtered bioinformatically to explore all of the genes linked to intellectual disability, autism and developmental conditions. This may reveal new diagnostic or prognostic information. Whilst it is unrealistic to suggest that all 20,000 genes will be analysed and reported as a whole ^8^, the resource is at least available to be interrogated as and when required. As genomic medicine is increasingly available across healthcare systems, we are moving closer to genetics becoming ‘mainstreamed’ ^7, 9^. Thus, patients have more exposure than ever before to the volume and complexity of genetic information.

While there are many ways in which genomics is improving healthcare, it is important to note that there is evidence that genomic medicine does not always simplify or improve care. For example Adam Hedgecoe ^10^ questions the utility of genetic testing, which can increase diagnostic uncertainty and Sophie Day *et al.*, ^11^ identify the ways in which genomic medicine further complicates treatment pathways for breast cancer. Arribas-Ayllon ^12^ reviews the literature to show that applications of genetic testing in healthcare are uneven and partial.

An increase in the scale of testing available inevitably also means an increase in the range of results returned – many of which, in the current climate, can only be interpreted as ambiguous or uncertain (due to the embryonic nature of our knowledge of this field) ^13^. Such ’Variants of Uncertain Significance’ are results where the meaning is unclear and are more likely to be discovered when multiple genes are tested for at once ^14^. In addition to the management of uncertainty, patients will be faced with more information options and so it becomes ever more pertinent to ask ‘how much do you really want to know?’ ^15^.

Given the ability to look at multiple genes within one test, genomic technologies deliver an opportunity to serendipitously explore genes unrelated to the health condition being explored. This means that when a patient has their cancer genes looked at it would also be possible, at the same time, to explore their genes linked to heart disease. Such results might be referred to as ‘additional looked for findings’ ^3^, ‘secondary or incidental findings’ or an ‘opportunistic screen’ ^15^. Indeed, within the NHS’s 100,000 Genomes Project parents of a child who is having genome sequencing can have the opportunity to be tested for ‘additional looked for findings’ related to their own risk of future disease, unrelated to their child’s condition ^16^.

As patients are exposed to increasingly complex and uncertain genetic information, debates have emerged as to the best way to manage this. Middleton *et al.*, ^15^ demonstrate that while many people are enthusiastic about receiving genetic information healthcare professions (especially those who work in genetics) are more cautious. Joon-Ho *et al.*, ^17^ suggest that genetic information should be framed as a ‘dynamic resource’ with uncertainty managed by viewing this information as a resource that should be revisited over the lifetime of an individual. There is as yet no consensus regarding the best way to manage the increasing volume of complexity and uncertainty generated by genomic sequencing. In the ongoing discussion we argue it is crucial that debates are informed by wide range of voices.

Genomic testing is also increasingly being used to guide treatment options and provide more individualised risk assessments, often called ‘precision’ or ‘personalised’ medicine ^18^. This allows clinicians and genetic counsellors to explore predispositions to developing future disease, thereby enabling steps for prevention, screening and/or management to be taken. Furthermore, this may be linked to pharmacogenomics – genetic testing used to guide drug use in medicine. For example, before prescribing, patients may be tested first to see if they are likely to be able to metabolise certain drugs ^19^. This is also being used in oncology, where chemotherapies are targeted towards people with certain genetic profiles ^20^.

Genomic information is different to other sorts of medical information, in that it is shared between biological relatives. So, even if a person is not using healthcare services themselves, they may be related to someone else who is; thus, the reach of the results moves outside of the clinical encounter and into the wider family. Whilst most of us will not currently be a patient in a healthcare setting, we may still be related to someone who is having testing and the questions they have answered may be very relevant to us too. Therefore, the impact of genomic information naturally extends beyond a healthcare encounter–through conversation it travels from the patient, out to their extended family and to people who are not yet patients. Such people could be considered a type of ‘patient in waiting’ ^21^; we don’t yet know where and how they seek out information and meaning, but it is likely they look to the media ^22^, popular culture ^23^ and the Internet ^24^ for insight. It is important when assessing how people respond to genetic information to ensure that ’the public’ is not envisioned as a singular, unified entity. Scholarship, especially from genetic counselling, demonstrates that the different ways people understand and make sense of genetic information is complex, varied and often idiosyncratic. ^25– 28^. An important ethical issue, especially in the context of continuing enthusiasm for genomics, is how to respect the wishes of those who dissent, turning down personal genomic knowledge ^28^.

The complex ways that genetics – and now genomics – will affect patients, means that the provision of genetic counselling is of increasing significance. Patients have voiced the opinion that the provision of genetic counselling is important when having genome sequencing ^29^. Researchers and clinicians have also highlighted the importance of drawing on the expertise of genetic counsellors in order to ensure new technologies are integrated appropriately into healthcare ^30^.

### People as consumers

Having discussed some of the implications of genetics for patients we will now explore some of the ways that genomics will affect people as ‘consumers.’ One way is via ‘direct to consumer’ (DTC) genetic testing. This is a growing industry with private companies marketing and selling a wide range of tests through the Internet. Consumers are able to send off a DNA sample (normally a saliva sample) and a few weeks later receive their test results. There are a wide range of DTC genetic tests both health related and non-health related. Health related tests include identifying predispositions to common and complex disorders, such as cancer and cardiovascular disease, tests for carrier status that could guide reproductive decisions, and nutrigenomics and pharmacogenomics that could potentially guide treatment and lifestyle choices ^31^. Non health-related tests include testing for paternity, ancestry, athletic ability, as well as traits such as earwax characteristics and caffeine metabolism ^31^. DTC genetic testing is rising in prominence. In 2008, 23andMe’s retail DNA testing kit was named invention of the year by Time magazine. In 2016, genomics was named by Forbes as one of the three ‘Big Technologies to Watch’ over the subsequent decade, together with nanotechnology and robotics, predicted to have the biggest impact on society ^32^.

The potential risks and benefits of DTC genetic testing have been widely debated. Proponents argue that they empower consumers to take responsibility for their health ^33^ and improve the quality of their life ^34^. However, researchers and health professionals have raised concerns about the clinical validity of DTC genetic tests, particularly in relation to susceptibility testing for complex disease, such as diabetes or dementia ^35, 36^. Specifically, researchers have argued that these tests fail to take into account non-genetic factors that can contribute to complex disease ^33^. For Mendelian conditions, where a single gene is causative of disease, concerns have been voiced regarding the negative and positive predictive value of DTC genetics tests ^37^. Researchers have also expressed concerns that when testing for serious, potentially life-threatening conditions, there is the possibility for unanticipated emotional reactions that cannot easily be addressed immediately via an Internet based service ^38^. Despite the professional bodies who represent genetic health professionals (e.g. European Society Human Genetics and American College of Medical Genetics and Genomics) recommending that genetic counselling should be provided by DTC genetic testing companies, a review of such services indicated that the support services on offer are often severely lacking ^39^.

DTC genetic testing is a growth market and as genetic testing becomes cheaper an increasing number of online genetic services will be available to members of the public ^40^. With purported plans to incorporate genomic testing into the Apple watch ^41^, as well as fitness monitoring market ^42^, we as consumers will be faced with more opportunities to engage with genomic technology and the implications for our health and wellbeing.

In making purchasing decisions consumers will increasingly have to draw on their scientific understanding as well as cultural beliefs in order to make purchasing decisions. Abrams and colleagues conclude:

"Such exposure to genomics information outside of the clinical setting call upon the public to independently evaluate the veracity of these claims and make related decisions" ^43^.

### People as citizens

So far, we have explored the implications of genetics for us as patients and consumers. Finally, by focussing on some of the novel ethical dilemmas that will arise from technological advancements, we are able to explore some of the broader societal implications of genomics for us as citizens.

Scholars have drawn attention to the ways in which commercial companies are exploiting new genomic technologies ^44^ with genomic and post-genomic (e.g. stem-cell) science seen as a crucial aspect of many state economic strategies ^45^. Indeed one of the primary functions of the 100,000 Genomes Project, the NHS genomic sequencing project, is: “Stimulating and enhancing UK industry and investment” ^16^.

The commercialisation of genomics is inevitable; indeed, to accelerate future drug development, it is imperative. However, with the for-profit industry comes an ethical tension in relation to the raw assets – genomes – that form the resource of data used in genomic research. Empirical research has shown us that whilst publics are willing to donate their DNA and medical information for use in research, they are more suspicious of donating their data for use by for-profit companies ^46^; with concerns that unaffordable medicines would be developed as well as profits made for shareholders that they would not benefit from. We are encouraged as citizens to be altruistic with the donation of our data for the greater good and yet at the same time many will benefit, in a monetary sense, from this altruism. This tension is subject to increasing debate ^47, 48^.

Genomic data is commonly stored electronically online; it is exchanged, shared and traded over the Internet every second of the day, as clinicians and researchers and pharmaceutical companies alike explore what a specific variant means. In order to interpret the significance of an individual finding, researchers need to see how this finding has expressed itself previously, by comparing it to datasets from thousands of other people ^39^. The importance of sharing data is outlined by Lucassen and Montgomery (2017) who say:

“Genomics offers benefits and responsibilities for the individual, the family, the broader community and globally that cannot be realized by keeping the secrets revealed from one genome separate from others.” ^49^(Ch16, p4)

Thus, for us as citizens there are now emerging ethical dilemmas in relation to data security and privacy. If the only way to fully realise the potential of genomics is to collect large volumes of genomic data from millions of people (to be accessed by clinicians, non-profit and for-profit researchers in the endeavour to better understand the link between genes and disease), then this means that such data needs to be stored online, shared, and protected against unauthorised use. Together with medical data, the storage of DNA information forms one of the key issues for international policy creation – with the pivotal issue being how to make online data storage safe and secure ^50^.

One very significant dataset of genomic information is contained within the UK’s National DNA Database. Here, DNA information is stored from people who are convicted, cautioned or recently arrested of a crime and can be matched to DNA collected from crime scenes. Scholars have raised concerns about how DNA should be utilised in criminal law, with DNA databases having a disproportionally high number of people of ethnic minority status ^51^. It was this fact that Lord Justice Stephen Sedley used when arguing for expansion of the National DNA Database. Sedley countered civil liberty concerns arguing that a universal database would be less discriminatory if it was representative of the national population ^51^.

Concerns about discrimination have also been raised in other policy areas regarding genetics. Scholars writing from a disability rights perspective have argued that genetic screening programs can reify a view of disability as purely a medical issue, ignoring social barriers that marginalise people in society. As such, implicit judgments are made about what is a worthwhile life, with genetic screening programmes having the potential to increase discrimination of people with disabilities ^52^.

## Conclusions

In this opinion article, we have charted some of the wide-ranging implications of advancements in genomics. The implications have been explored in relation to the experience people have in three contexts, as patients, consumers and citizens.

Given that genomic technology is here to stay and is increasing its foothold across the whole of society, we need to be mindful of taking stock and reviewing what sort of society we want to live in. There needs to be more psychosocial research to understand the attitudes, values and opinions people have about the use and application of genomics. Without this we are in danger of the technology being prescriptive of how society should function, instead of the other way around. This can happen when technological advances lead to what *can* be done being conflated with what *should* be done. For example, with prenatal screening programs there is evidence that as new technologies become routinized they have driven medical practice ^53^. This happens when the ‘normal’ thing to do has become conflated with the ‘right’ thing to do. Scholars writing from a disability rights perspective have noted that this may subvert the wishes of patients who may not want screening ^54, 55^. In a medical setting - where there are complex power dynamics in play - patients may not feel empowered to state a preference that goes against what is construed as ‘normal’ and therefore ‘right’.

The societal implications of genomics include many facets. Within this article we provide a brief overview of the availability of genomic tests within medicine and the subsequent increase in engagement with uncertainty, the Direct to Consumer genetic testing market and commercialisation of genomics, the use of DNA databases by the police, the concerns about genomic screening programmes propagating discrimination and the necessity to ensure privacy and security of genomic data. These are all examples of the ways in which genetics/genomics will influence our lives as patients, consumers and citizens, and are all areas that are important for policy consolidation.

Genomic technology is now so pervasive across society and all of us, whether we see ourselves as a patient (or biologically related to a patient), a consumer or a citizen, we are likely to be confronted by the outcomes of genomics. Because of this there is an urgent need to explore the impact of the technology from many different societal perspectives. Together with normative bioethics, empirical data from people making sense of genomics should guide policy decision making so that the implementation of genomic technology is a positive endeavour that benefits humankind.
