# Supplementary material for: Genetics in the 21st Century: Implications for patients, consumers and citizens
Source: F1000Res. 2018 Jan 24;6:2020. Originally published 2017 Nov 17. [Version 2] doi: 10.12688/f1000research.12850.2 (PMC5721930; doi:10.12688/f1000research.12850.2)
Supplement: Peer review history

# Peer review: Referee response for version 2

**DOI:** 10.5256/f1000research.14992.r30163

**Published:** 2018-02-19

**Authors:** Lorraine Cowley

**Affiliations:** 1 Newcastle University, Newcastle upon Tyne, UK

I accept the authors’ responses to the comments made and I am happy to approve the amended article.

I have read this submission. I believe that I have an appropriate level of expertise to confirm that it is of an acceptable scientific standard.

# Peer review: Referee response for version 2

**DOI:** 10.5256/f1000research.14992.r30162

**Published:** 2018-02-05

**Authors:** Michael Arribas-Ayllon

**Affiliations:** 1 Cardiff School of Social Sciences, Cardiff University, Cardiff, UK

The paper is much improved and am persuaded by their responses. I am satisfied that the Opinion Paper is ready for indexing.

I have read this submission. I believe that I have an appropriate level of expertise to confirm that it is of an acceptable scientific standard.

# Peer review: Referee response for version 1

**DOI:** 10.5256/f1000research.13925.r28119

**Published:** 2017-12-07

**Authors:** Tina-Marie Wessels

**Affiliations:** 1 Division Human Genetics, University of Cape Town, Cape Town, South Africa

This opinion piece covers the pertinent issues we are currently grappling with as genomic medicine gains momentum. The authors highlighted the many facets and far-reaching consequences well.

 The authors discussed how we have moved from single genes to panels (many genes) to whole exome and genome sequencing and the use of bioinformatics to identify relevant variants. An important issue is the different tools available in bioinformatics, how these are applied in specific cases and how these effect what is regarded as significant and what is not. This field is growing and it will continue to improve its algorithms so to increase the accuracy of variant calling. In addition, distinguishing between normal variation and pathogenicity is currently problematic when working with non-European populations. Concerted affords are underway to address the underrepresentation of diverse population (Ramesar 2015 ^1^).

 The authors state that genetic counselling will be increasingly more significant. It is important to mention that the role of clinical genetics i.e. taking a comprehensive family history and using clinical information to guide and or assist with the interpretations of genomic data will remain important. Except for the traditional role of genetic counsellors helping consumers understand and make informed choices as pointed out, the role of genetic counsellors is expected to expand to include providing support to other healthcare providers involved with the ordering of genomic tests, assistance in developing online tools and education for both professionals and the public.

 One aspect that requires more consideration in the genomics era is the need for education. The authors quote Abrams and colleagues that highlight the need for public education so that consumers can make informed decisions. Education for the public is essential but there is also a need for education of medical and other health care professionals as they will increasingly be involved in ordering and or interpreting genomic tests and use these results in the care of their patients. There are so many tests to choose from which makes it difficult to evaluate which have clinical utility.

 The authors highlighted the ethical dilemmas related to data security and privacy. Ethical dilemmas have been highlighted since the start of the human genome project (ELSI). The issues related to incidental findings and informed consent could be included in the article.

I have read this submission. I believe that I have an appropriate level of expertise to confirm that it is of an acceptable scientific standard.

## References

[1] RamesarR: Genomics: African dawn. *Nature*.2015;517(7534) : 10.1038/nature14077 276-7 10.1038/nature14077 25470066

# Peer review: Referee response for version 1

**DOI:** 10.5256/f1000research.13925.r28116

**Published:** 2017-12-07

**Authors:** Michael Arribas-Ayllon

**Affiliations:** 1 Cardiff School of Social Sciences, Cardiff University, Cardiff, UK

This is a clear and well-structured opinion piece that identifies many relevant issues concerning genetic and genomic testing and its presumed impact on society. I am unfamiliar with this journal, so I am unsure how to gauge the usefulness of this article for its readership.

 My main concern is the extent to which the authors claim the pervasiveness of genomic technologies. In the sociological literature, with which I am more familiar, these claims are not all clear or straightforward. For instance, Adam Hedgecoe questions the utility of genetic testing, which can increase diagnostic uncertainty (also see Anne Kerr’s work). He also argues that in the context of pharmacogenomics, genomic medicine *in the clinic* falls short of personalised medicine. Arribas-Ayllon (2016) reviews the literature to show that applications of genetic testing in healthcare are uneven and partial. Sophie Day et al. (2016) has also identified the ways in which genomic medicine further complicates treatment pathways for breast cancer. What is common to these and many other sociological studies is that genomic medicine consistently fall short of the hype that genetic and genomic technologies will be revolutionary and personalizing.

 For instance, I am troubled by the frequent use of the pronoun ‘all’ in the first paragraph to suggest that genomic medicine is all-encompassing and totalising. It is not at all clear to me that genomic medicine is rolled out as smoothly and as evenly as the authors suggest. In fact, I wonder, given the age of austerity we now live under, whether these technologies will live up to the hype of delivering a ‘truly’ personalised medicine. I also wonder about the circumstances and sites in which this technology is not routinely available to everyone.

 I am also troubled by the assertion made at the end of the third paragraph that genomic medicine ‘has been truly ‘mainstreamed’’. This is a strong statement. What confidence do the authors have that this is actually true? Of course, I think it serves the authors’ argument to confidently state that genomic medicine has arrived and is here to stay because it justifies the urgent need to foster public dialogue about ethical and social implications regarding complex health information. But I feel the authors are overstating these claims. Again, sociological studies maintain that the roll-out of these technologies is not even nor does it not necessarily simplify or improve treatment pathways.

 In so far as genomic medicine gives out complex and uncertain information, I think the author would benefit from reading the work of Joon-Ho et al. (2013) to elaborate the kinds of issues that arise when ‘returning results’ is framed in terms of a ‘dynamic resource’ of information that needs to be managed over the lifetime of an individual. This also has unique implications for genetic counselling.

 Although I understand the authors are discussing genetics in general, the example of DTCGT is based on a different technology to that of the previous examples concerning whole genome sequencing. DTC companies use *genotyping* to calculate probabilistic risk for common complex disorders. I think the technical differences between sequencing and genotyping ought to be flagged. The general reader might reach the opinion that all genome testing is based on the same technologies.

 In their Conclusion, the authors claim that there is a danger that genomic technology will be ‘prescriptive of how society should function, instead of the other way around’. Firstly, I don’t think this point has been sufficiently developed in the paper, or at least it doesn’t follow naturally from the arguments and examples given above. I am also concerned that because the author are buying into the revolutionary hype of genomic medicine, that society is construed as being essentially passive.

**Recommendations**

 I recommend that the authors tone down their claims that genomic medicine is revolutionary and pervasive.

 I recommend the authors indicate that there are still significant obstacles of delivering personalised and stratified medicine in real-world settings.

 I recommend that the authors explore more deeply the kind of relationship individuals can expect from sequence-driven information, especially in relation to how this information is likely to change over time (see Joon-Hu et al. 2013 ^1^).

**Minor revisions:**

 On the last line of the second column on page 2 ‘where chemotherapies are targeted towards a people with…’ should read ‘where chemotherapies are targeted towards people with…’

 On page 3, first column, in the second paragraph 2 ^nd^ line ‘provision of genetic counselling is of increasing in significance’ should read ‘‘provision of genetic counselling is of increasing significance’.

I have read this submission. I believe that I have an appropriate level of expertise to confirm that it is of an acceptable scientific standard, however I have significant reservations, as outlined above.

## References

[1] YuJHJamalSMTaborHKBamshadMJ: Self-guided management of exome and whole-genome sequencing results: changing the results return model. *Genet Med*.2013;15(9) : 10.1038/gim.2013.35 684-90 10.1038/gim.2013.35 23619276PMC4010112

# Response

**Published:** 2018-01-18

**Authors:** Jonathan Roberts

**Affiliations:** Wellcome Trust Sanger Institute, UK

Thank you very much to Michael for his review. We have addressed his comments below (Reviewers comments are in italics) 

*This is a clear and well-structured opinion piece that identifies many relevant issues concerning genetic and genomic testing and its presumed impact on society.*

 Thank you.

*I am unfamiliar with this journal, so I am unsure how to gauge the usefulness of this article for its readership.*

 We chose F1000 because we wanted a platform with good search engine optimisation, so that our article would be very easy to access and find (and thus not be limited by a single journal authorship group). The article has had 364 reads in a matter of weeks, so this bodes well in terms of reaching an interested audience. The paper was written as a broad, light-touch opinion piece as opposed to addressing a specific issue for a particular audience, hence choosing a generic journal that covers all scientific disciplines. We also wrote it for a non-specialist, generic audience.

*My main concern is the extent to which the authors claim the pervasiveness of genomic technologies.*

 There is absolutely no doubt that genomic technologies are here to stay and are embedded in many different settings. Our reference to the Chief Medical Officers report on Genomics provides evidence to the commitment to further embed genomic technology into healthcare services in the UK. We have also provided evidence (page 3, column 1, ‘people as patients), outside of the UK where genomic technologies are being applied, on a large scale, for example in Australia, Qatar and the USA. In the interests of space we chose not to list the many other global sequencing initiatives, but for the reviewer’s information, we could have added in details about the national sequencing initiatives in Pakistan, Switzerland, Canada, Turkey, Singapore, India, China, Japan and Latin America to name but a few countries to evidence the global reach of genomic technologies.

 We also draw the reviewer’s attention to the ‘Million European Genomes Project’, where genomic and health data will be linked from 1 million European patients: [Horgan D, Romao M, Hastings R. Pulling the Strands Together: MEGA Steps to Drive European Genomics and Personalised Medicine. Biomedicine Hub. 2017;2(suppl 1)481300 (DOI:10.1159/000481300)]. And also to the work of the Global Alliance for Genomics and Health ( www.ga4gh.org) which represents more than 500 organisations worldwide all involved in genomic sequencing initiatives.

 We have added some text about global utilisation of genomics: “A commitment to significant and ongoing investment in genomics on an international scale is demonstrated by the ‘Million European Genomes Project’, where genomic and health data will be linked from 1 million European patients  and The Global Alliance for Genomics and Health ( www.ga4gh.org)  that represents more than 500+ organisations globally all with the shared endeavour of implementing and enabling genomic technology to be applied in research and clinical practice”

*In the sociological literature, with which I am more familiar, these claims are not all clear or straightforward. For instance, Adam Hedgecoe questions the utility of genetic testing, which can increase diagnostic uncertainty (also see Anne Kerr’s work).*

 Whilst it is true that the implementation of genomic medicine brings with it many uncertainties (and we have clearly alluded to some of these on page 3, second column, second para) we would contest that that genomic technology is pervasive and being delivered on a large scale.  

*He also argues that in the context of pharmacogenomics, genomic medicine in the clinic falls short of personalised medicine. Arribas-Ayllon (2016) reviews the literature to show that applications of genetic testing in healthcare are uneven and partial.*

 We are not in disagreement with the reviewer about the difficulties that genomic technologies bring and that the application across healthcare settings is uneven. Audiences could be guided to our other publications that offer more of a reflection of what is working and the difficulties, particularly for genetic counsellors, in the application of genomics in a healthcare setting, e.g. : Middleton A, Hall G, Patch C (2015) Genetic counsellors and Genomic Counselling in the United Kingdom. Molecular Genetics and Genomic Medicine 3(2) 79-83.)

 Instead the purpose of the Opinion piece was to offer a non-specialist audience a summary piece on the different ways genomics will touch us as patients (or related to patients), citizens and consumers.

*Sophie Day et al. (2016) has also identified the ways in which genomic medicine further complicates treatment pathways for breast cancer. What is common to these and many other sociological studies is that genomic medicine consistently fall short of the hype that genetic and genomic technologies will be revolutionary and personalizing.*

 We don’t disagree with this. But it was not the aim of the Opinion Piece to offer a critique of these issues. Neither was it the aim of the Opinion Piece to provide copious examples of where genomic medicine is working incredibly well (of which there are many). However, it is important to ensure that this article acknowledges the implementation of genomics is complex. As such we have added the following text at page 3, column 1, paragraph 3

 “While there are many ways in which genomics is improving healthcare, it is important to note that the implications of genomic medicine are multifaceted. For example Adam Hedgecoe (2008) questions the utility of genetic testing, which can increase diagnostic uncertainty and Sophie Day et al., (2017) has identified the ways in which genomic medicine further complicates treatment pathways for breast cancer. Arribas-Ayllon (2016) reviews the literature to show that applications of genetic testing in healthcare are uneven and partial.”

*For instance, I am troubled by the frequent use of the pronoun ‘all’ in the first paragraph to suggest that genomic medicine is all-encompassing and totalising. It is not at all clear to me that genomic medicine is rolled out as smoothly and as evenly as the authors suggest. In fact, I wonder, given the age of austerity we now live under, whether these technologies will live up to the hype of delivering a ‘truly’ personalised medicine. I also wonder about the circumstances and sites in which this technology is not routinely available to everyone.*

 In response to the above comment we have ‘toned down’ some of the language. Specifically:

 In the abstract, paragraph 1, line 8: “‘personalised medicine’ is now a reality”  now reads    “‘personalised medicine’ is now moving closer to a reality.”

 In the abstract, paragraph 2, line 2: “The impact of such a ‘genomic era’ is likely to have some level of impact on all of us”, now reads:    

 “The impact of such a ‘genomic era’ is likely to have some level of impact on an increasingly large number of us”

*I am also troubled by the assertion made at the end of the third paragraph that genomic medicine ‘has been truly ‘mainstreamed’’. This is a strong statement. What confidence do the authors have that this is actually true?*

 In response to this comment some of the language has been changed. In page two, paragraph four, the penultimate sentence “Genomic medicine is now available across whole healthcare systems, it has been truly ‘mainstreamed’” now reads:

 “As genomic medicine is increasingly available across healthcare systems, we are moving closer to genetics becoming ‘mainstreamed’”

 While the author raises a valuable point regarding mainstreaming, it is undeniable that genetics is increasingly being used across a range of healthcare settings. For example at the time of writing this comment (17/01/18) research has been published in the Journal of the National Cancer Institute that has provided evidence that BRCA testing for all women over the age of 30 is cost effective.

 The Society and Ethics Research Group (where the authors are from) are part of the Wellcome Genome Campus in Cambridge, which in turn is a partner in the East of England Genomic Medicine Centre. Through teaching NHS members of staff, funded via Health Education England on the new MSc Genomic Medicine at the University of Cambridge, the authors are in contact with NHS staff involved in ‘mainstreaming’ genomics (i.e. applying new genomic knowledge to their practice). It is this experience that informs our knowledge of the way genomic technologies are influencing practice outside of specialist clinical genetics services. Thus we have confidence and experience in the statements we have made about genomics in a healthcare setting.

*Of course, I think it serves the authors’ argument to confidently state that genomic medicine has arrived and is here to stay because it justifies the urgent need to foster public dialogue about ethical and social implications regarding complex health information. But I feel the authors are overstating these claims. Again, sociological studies maintain that the roll-out of these technologies is not even nor does it not necessarily simplify or improve treatment pathways.*

 We do not believe the article implied that these technologies are simplified or have improved pathways in all situations. However, there is significant evidence that genomics has improved care in number of settings. Notably, in rare disease and the diagnosis of developmental delay, genomics has made it less likely that patients will have a long ‘diagnostic odyssey’. Importantly for our purposes here, our argument is not based on genomics universally improving care. Rather that genomics will be increasingly used in healthcare settings. This may improve care (such as in rare disease) or may lead to increased uncertainty with a higher likelihood of finding and Variants of Uncertain significance. The key point is that dialogue needs to be fostered because genomics will have increasingly large applications in healthcare, a claim that is well supported through empirical evidence.       

 For examples of genomics improving care see the following reference for details of the cost effectiveness of sequencing in a clinical setting: Walsh et al (2017)  The reviewer is also directed to the work of the Deciphering Developmental Disorders project that now has a diagnostic rate of more than 40%, i.e. for children with severe developmental disorders who have not been able to receive a diagnosis for their condition via traditional NHS testing, more than 40% have received their diagnosis after exome sequencing. The DDD project also predicts that if whole exome sequencing was offered to these children as a first line test, more than 50% would have received a diagnosis for their condition : Wright et al (2018)

*In so far as genomic medicine gives out complex and uncertain information, I think the author would benefit from reading the work of Joon-Ho et al. (2013) to elaborate the kinds of issues that arise when ‘returning results’ is framed in terms of a ‘dynamic resource’ of information that needs to be managed over the lifetime of an individual. This also has unique implications for genetic counselling.*

 We did not want to engage in lengthy theoretical debate about the best ways complexity and uncertainty can be managed in clinical settings (this is a paper in itself!) We have already published our own empirical work (n = 7,000) on the return of results from sequencing studies [see Middleton et al 2016]. We are aware of Joon-Ho Yu et al’s excellent work. However, as this is based on a theoretical approach that is not being applied within clinical settings we feel it is perhaps premature to offer this as a suggestion for how genomic medicine is working in practice now. However, the reviewer draws attention to an important debate. As such we have added the following text at page 3, column 1,  penultimate paragraph. .

 “As patients are exposed to increasingly complex and uncertain genetic information, debates have emerged as to the best way to manage this. Middleton et al., demonstrate that while many people are enthusiastic about receiving genetic information health professions (especially those who work in genetics) are more cautious. Joon-Ho et al., suggest that genetic information should be framed as a ‘dynamic resource’ with uncertainty managed by viewing this information as a resource that should be managed over the lifetime of an individual. There is as yet no consensus regarding the best way to manage the increasing volumes of complexity and uncertainty generated by genetic sequencing. In the ongoing debates we argue it is crucial that debates are informed by wide range of voices.”       

*Although I understand the authors are discussing genetics in general, the example of DTCGT is based on a different technology to that of the previous examples concerning whole genome sequencing. DTC companies use genotyping to calculate probabilistic risk for common complex disorders. I think the technical differences between sequencing and genotyping ought to be flagged.*

 DCT also look for SNPs in genes that cause mendelian disease, the key issue here is their positive/negative predictive value.

*The general reader might reach the opinion that all genome testing is based on the same technologies.*

 We understand the concern regarding the confusion of what could be called ‘whole genome sequencing’. This term could refer to a wide range to technologies (micro-arrays, SNP arrays, whole exome sequence). We believe we have covered the key concerns of DTCGT in this section (clinical validity for information about common diseases and positive and negative predictive values for mendelian disease). We feel that additional information regarding the specific technologies would not add clarity. However, we have added a short explanation at the beginning on the paper clarifying how we are utilising the term ‘whole genome’ and that this references to a range of different technologies that provide different levels of information.

 We have added the following text in at the beginning of the article (before “people as patients” section).

 Before the continuing it is important to make clear that in this article we use the term ‘genomic’ to refer to a wide range of sequencing and laboratory technologies. There are a range of different technologies that interrogate the ‘whole genome.’ Next generation sequencing may be used to create a ‘whole genome sequence’  - a complete sequence all ~ 3 billion base pairs of an individual's genome. However to generate a true whole genome sequence multiple sequencing methods (other than NGS) may need to be used.  Furthermore there are a number of technologies that look across an entire genome but do not create a ‘whole genome sequence’ for example, microarrays, SNP arrays, and exome sequences. For our purposes we refer to all technologies that look across the genome  as ‘genomic’. Where there are specific technological issues (such as positive and negative predictive values in direct to consumer genetic tests) we refer to these. However, for clarity, we do not feel it it would be beneficial in this article to expand on the different technologies used for sequencing in different settings. As such for purposes of this article we discuss broadly the implications issues raised by ‘genomic’ technology with an understanding that this refers to a range of sequencing and testing techniques.                

*In their Conclusion, the authors claim that there is a danger that genomic technology will be ‘prescriptive of how society should function, instead of the other way around’. Firstly, I don’t think this point has been sufficiently developed in the paper, or at least it doesn’t follow naturally from the arguments and examples given above. I am also concerned that because the author are buying into the revolutionary hype of genomic medicine, that society is construed as being essentially passive.*

 The point is to highlight the fact that different publics’ views need to be taken in account as genomic technology becomes increasingly relevant in a range of settings. Whilst we would not want to construe the public as passive, we must not ignore the importance of power relationships and how in discourses of scientific and medical expertise ‘non- expert’ voices can be ignored. We believe that while it is important to value expertise, we must also ensure that there is dialogue and that non-expert voices do not become marginalised. This is why research into the attitudes and opinions of different publics is so valuable.

 We would also point out that there are empirical examples of how new technologies can become prescriptive of the way society functions. This happens when what *can* be done is conflated with what *should *be done.  For example, with prenatal screening programs, there is evidence that as new technologies become routinised they have driven medical practice. This happens when the ‘normal’ thing to do has became conflated with the ‘right’ thing to do. Scholars writing from a disabilities rights perspective have noted that this may subvert the wishes of patients who may not want screening. In a medical settings - where there are powerful power dynamics in play - patients may not feel empowered to state a preference that goes against what is ‘normal’ and ‘right’. It is this we refer to when we say that technology may become prescriptive and we have added some text to further this argument on page 5, paragraph 2 of ‘conclusions’ section.

**Recommendations**

*I recommend that the authors tone down their claims that genomic medicine is revolutionary and pervasive.*

 We hope we have managed to re-defend our view that genomics is indeed pervasive across society. We have, however, toned down our claims as requested.

*I recommend the authors indicate that there are still significant obstacles of delivering personalised and stratified medicine in real-world settings.*

 We have added reference to the obstacles to delivering personalised medicine, however, we have not elaborated in detail on these as this wasn’t the focus of the Opinion Piece and we wanted to keep the piece deliberately broad.

*I recommend that the authors explore more deeply the kind of relationship individuals can expect from sequence-driven information, especially in relation to how this information is likely to change over time (see Joon-Hu et al. 2013* *^1^* *).*

 We feel this could form a detailed discussion in a different paper (postulating on the possible futures that could exist with different applications of genomic medicine); however, Joon-Hu et al’s theoretical debate about the relationship between person and their data, is not being applied in practice at the moment and thus we do not feel it should form a significant part of our current Opinion Piece.

**Minor revisions:**

*On the last line of the second column on page 2 ‘where chemotherapies are targeted towards a people with…’ should read ‘where chemotherapies are targeted towards people with…’*

 Corrected

*On page 3, first column, in the second paragraph 2* *^nd^* *line ‘provision of genetic counselling is of increasing in significance’ should read ‘‘provision of genetic counselling is of increasing significance’.*

 Corrected

# Peer review: Referee response for version 1

**DOI:** 10.5256/f1000research.13925.r28118

**Published:** 2017-12-06

**Authors:** Lorraine Cowley

**Affiliations:** 1 Newcastle University, Newcastle upon Tyne, UK

The scope of this review does not include proof-reading; I have not commented on grammatical or typographical errors.

 The authors have written a timely and relevant opinion piece which should be welcomed by the genetic counselling community. The article aims to explore how we might experience advancements in genomic technology as patients, consumers and citizens and urges policy makers to take stock of the human response to and pervasiveness of genomics.  I strongly agree with the authors’ conclusions that genomics is a current and important topic for society and in need of further psychosocial research. However, I would ask the authors to consider further illustrating and discussing the points made in the conclusion throughout the article.

 As a general point, it would be helpful to have a brief statement about the academic disciplinary background of the authors to give the reader a better understanding about the context of the article and an idea about the lens through which their opinions are given. 

 Perhaps the potted history of the emergence of genomic technology in the abstract would sit better in the introduction with some comment from the authors on how the availability and reduced cost of technology is influencing its application. Some overstatement maybe avoided, for example, the countries they quote where genomic technology is being used outside the UK do not suggest global utilisation and maybe using alternative phrasing such as the ‘economically developed world’ would reflect the situation more accurately. As this is an opinion piece, it would have been good to see more critical engagement with the literature that the authors have cited and more of their opinion. Perhaps each quotation cited could be followed by comment from the authors; whether they contest, agree in part, find problematic or endorse what has been said. It would be helpful to have a brief explanation of their understanding of certain terms and concepts, as there are often inter and intra-disciplinary differences in definitions, for example, it would be helpful to know what their understanding is of  ‘normative bioethics’. It is welcome to see mention of the disability rights perspective and issues of potential discrimination with references to social science literature. Given that the opinion piece calls for a societal review, discusses sociological constructs such as citizenship and outlines current applications of genomic/genetic technologies, maybe it would be improved by further engagement with social science literature.

 The authors aim to explore the human response to genetic advancements in genetics. A large proportion of the section headed ‘People as patients’ seems focused more on the technological capacity to offer tests and generate information rather than the human response to those technologies.

 They make the point well about the shared nature of genetic information but stop short of acknowledging that not everyone has the same approach to knowing this information. The point could be enhanced by relating the discussion to the wealth of genetic counselling research that explores the communication of genetic risk in families, thus considering the implications for patients diagnosed with genetic conditions and those who do and do not want to know. I feel that this is a missed opportunity, if not to explore in detail, given the length of the article, at least to mention how finding out genetic information through mainstream diagnostic tests and/or direct to consumer (DTC) genetic testing may influence the communication dynamics within families about genetic risk and what this might mean to people. The authors seem to have assumed normativity in how genetic/genomic information is cascaded throughout families to ‘patients in waiting’ when in contemporary genetic counselling practice we know that this can be complex and challenging.

 The article would be enhanced by either making explicit their stance towards the current and future applications of genomics which they have highlighted, or identifying authors who contest or problematize the issues. For example, but not limited to, it would be interesting to know their opinions on:

 Do they uncritically accept that genomics poses responsibilities (Lucassen and Montgomery, 2017) for individuals and what do they understand those responsibilities to be?

 What place do they think there is for dissenters or test decliners (if any) in the future genomic society?

 What do they think about mainstreaming?

 What do they think about the current and purported future use of DTC genetic testing or the issues this currently raises in genetic counselling?

 What do they make of the claims about the usefulness of genetic information in, for example, the fitness industry?

 Do they believe that people are equipped to make distinctions about the veracity of claims?

 Is there a contrasting point to be made in how clinicians interpret the clinical significance of (make distinctions about) genomic information and translate that into clinical care?

 The authors flag the important idea that genomic testing may create uncertainty and this point could be enhanced by critically discussing the ways in which genomics dispels and confirms uncertainty and the limitations of what can be known versus what can be done.

 Addressing these issues would go some way to exploring why policy-makers should approach the pervasiveness of genetics with caution. In summary, I believe the authors could state and argue more clearly the limitations to claims that genomics potentially benefits our health, our lifestyle and our cultural practices.   

 Given further critical engagement with these arguments, the opinion article could provide a strong overview of nascent genomics in the early twenty first century. This is a really useful article by leading and emerging researchers in the genetic counselling field and it would be beneficial to hear the authors’ opinions more explicitly stated.

I have read this submission. I believe that I have an appropriate level of expertise to confirm that it is of an acceptable scientific standard, however I have significant reservations, as outlined above.

# Response

**Published:** 2018-01-18

**Authors:** Jonathan Roberts

**Affiliations:** Wellcome Trust Sanger Institute, UK

Thank you very much to Lorraine for her review. We have addressed her comments below (with reviewer comment in italics). As a broad response to the reviewer’s comments we would thank her for thoughtful feedback. We accept that the reviewer draws attention a number of important debates. We would stress that the purpose of this article is to highlight, in a broad way, the myriad implications of genetics (and hence the need for continuing sociological research) without getting drawn into any specific ethical debates in detail.

*The scope of this review does not include proof-reading; I have not commented on grammatical or typographical errors.*

*The authors have written a timely and relevant opinion piece which should be welcomed by the genetic counselling community.*

 Thank you. While we hope the article will appeal to the genetic counselling community our main hope is that it will be read by a broad audience who may be interested in the future of genetics.  

*The article aims to explore how we might experience advancements in genomic technology as patients, consumers and citizens and urges policy makers to take stock of the human response to and pervasiveness of genomics.  I strongly agree with the authors’ conclusions that genomics is a current and important topic for society and in need of further psychosocial research. However, I would ask the authors to consider further illustrating and discussing the points made in the conclusion throughout the article.*

*As a general point, it would be helpful to have a brief statement about the academic disciplinary background of the authors to give the reader a better understanding about the context of the article and an idea about the lens through which their opinions are given.*

 I’m afraid we are restricted to the editorial policies of the journal and so unable to address this.

*Perhaps the potted history of the emergence of genomic technology in the abstract would sit better in the introduction with some comment from the authors on how the availability and reduced cost of technology is influencing its application.*

 We feel this is covered in the opening paragraph and we believe that an extended history would not be warranted for a short opinion article.

*Some overstatement maybe avoided, for example, the countries they quote where genomic technology is being used outside the UK do not suggest global utilisation and maybe using alternative phrasing such as the ‘economically developed world’ would reflect the situation more accurately.*

 We agree that it is important not to overstate the implementations of genetics. However, we feel it is important to stress that genomics is a global venture.  Indeed the Global Alliance for Genomics and Health, representing 500+ organisations across the world, all involved in some way in the implementation of genomics, including in third world countries.

 We have added  some text to further clarify this on page 2, section “people as patients” paragraph 2.

*As this is an opinion piece, it would have been good to see more critical engagement with the literature that the authors have cited and more of their opinion. Perhaps each quotation cited could be followed by comment from the authors; whether they contest, agree in part, find problematic or endorse what has been said. It would be helpful to have a brief explanation of their understanding of certain terms and concepts, as there are often inter and intra-disciplinary differences in definitions, for example, it would be helpful to know what their understanding is of  ‘normative bioethics’. It is welcome to see mention of the disability rights perspective and issues of potential discrimination with references to social science literature. Given that the opinion piece calls for a societal review, discusses sociological constructs such as citizenship and outlines current applications of genomic/genetic technologies, maybe it would be improved by further engagement with social science literature.*

 We welcome the comments regarding the need for a critical engagement with the literature. The aim and scope of this article was to provide an overview of the wide ranging implications of genomics and as such to draw attention to the need for ongoing research of the type that the reviewer mentions. The article is brief and written for a general audience therefore we do not feel we need to describe what our understanding of certain terminology, e.g. ‘normative bioethics’.     

*The authors aim to explore the human response to genetic advancements in genetics. A large proportion of the section headed ‘People as patients’ seems focused more on the technological capacity to offer tests and generate information rather than the human response to those technologies.*

 We think the reviewer highlights important work, i.e. that which explores the human response to technologies. The aim of this paper was to provide a broad, accessible overview of the implications of genomics and as such to highlight the need for such sociological work. We feel that exploring in detail the sociological literature would be beyond the scope of this paper.  

   

*They make the point well about the shared nature of genetic information but stop short of acknowledging that not everyone has the same approach to knowing this information. The point could be enhanced by relating the discussion to the wealth of genetic counselling research that explores the communication of genetic risk in families, thus considering the implications for patients diagnosed with genetic conditions and those who do and do not want to know. I feel that this is a missed opportunity, if not to explore in detail, given the length of the article, at least to mention how finding out genetic information through mainstream diagnostic tests and/or direct to consumer (DTC) genetic testing may influence the communication dynamics within families about genetic risk and what this might mean to people. The authors seem to have assumed normativity in how genetic/genomic information is cascaded throughout families to ‘patients in waiting’ when in contemporary genetic counselling practice, we know that this can be complex and challenging.*

**Possible new text:**

 We think the reviewer raises an important point. This paper primarily focuses on the implications of technological advances and the need for ongoing sociological research. However, it is also important to note the rich vein of scholarship, especially that from genetic counselling, that explores the ways people understand and make sense of genetic information. We have added some text - page 3 column 2 paragraph 2 - to draw readers attention to this scholarship.  

*The article would be enhanced by either making explicit their stance towards the current and future applications of genomics which they have highlighted, or identifying authors who contest or problematize the issues.*

 We appreciate the desire for a more explicit stance towards the applications of genomics.  However we feel that to do this with such a broad overview of the topic has the potential to triple the length of the paper.  As such we have not provided a thorough discussion about additional issues. This being said, the reviewer raises some important points and we have added some text to the paper that acknowledges the authors who have discussed problematic issues that have arisen regarding the implications of genetics. This can be found at page 3, column 1, paragraph 3

*For example, but not limited to, it would be interesting to know their opinions on:*

*Do they uncritically accept that genomics poses responsibilities (Lucassen and Montgomery, 2017) for individuals and what do they understand those responsibilities to be?*

 Whether there are indeed responsibilities imposed by genomics and how these are handled by organisations, such as the NHS, could form the basis of a whole new paper and we do not feel we can do justice to this topic within the confines of a sentence or two.

*What place do they think there is for dissenters or test decliners (if any) in the future genomic society?*

 We have added a sentence to draw readers attention to the important debate with respect dissenters or test decliners.page 3 column 2 paragraph 2, final line.

*What do they think about mainstreaming?*

 Mainstreaming is such a large topic that it is difficult to summarise this within a sentence or two; we feel this is outside the scope of a broad overview piece. We have also published separately on the emerging roles for genetic counsellors in genomic medicine and the impact of mainstreaming, see Middleton et al

*What do they think about the current and purported future use of DTC genetic testing or the issues this currently raises in genetic counselling?*

 We have separately published on this issue, see Middleton et al 2016

*What do they make of the claims about the usefulness of genetic information in, for example, the fitness industry?*

*Do they believe that people are equipped to make distinctions about the veracity of claims?*

*Is there a contrasting point to be made in how clinicians interpret the clinical significance of (make distinctions about) genomic information and translate that into clinical care?*

 These are all interesting and valid questions. The aim of the paper is to draw attention to the pertinence of these questions, to show the need for continuing research rather than to provide concrete answers or a comprehensive review of the sociological literature. The relationship between different publics, expertise and genetic information is the subject of the author’s PhD and publications detailing this research will be forthcoming.   

*The authors flag the important idea that genomic testing may create uncertainty and this point could be enhanced by critically discussing the ways in which genomics dispels and confirms uncertainty and the limitations of what can be known versus what can be done.*

 Research, especially from genetic counselling, has explored some of the complex ways that patients deal with uncertainty. The potential for increasing levels of uncertainty in genomics provides impetus for further research into how patients cope with, are resilient to and understand uncertainty. We have added some text at page 3, column 1, penultimate paragraph to draw readers attention to the complex debate surrounding uncertainty.    

*Addressing these issues would go some way to exploring why policy-makers should approach the pervasiveness of genetics with caution. In summary, I believe the authors could state and argue more clearly the limitations to claims that genomics potentially benefits our health, our lifestyle and our cultural practices.*

 The reviewer raises important points regarding the potential benefits and limitations of genetics. Within the confines of this short opinions piece we have added texts that further draws attention to this important debate.

*Given further critical engagement with these arguments, the opinion article could provide a strong overview of nascent genomics in the early twenty first century. This is a really useful article by leading and emerging researchers in the genetic counselling field and it would be beneficial to hear the authors’ opinions more explicitly stated.*

# Peer review: Referee response for version 1

**DOI:** 10.5256/f1000research.13925.r28114

**Published:** 2017-11-27

**Authors:** Anna Michell

**Affiliations:** 1 Division of Cardiovascular Medicine, Wellcome Trust Centre for Human Genetics, University of Oxford, John Radcliffe Hospital, Oxford, UK

The authors provide an overview of the current state of use of genomic technologies in the UK, comparing this with other nations as well as considering the wider implications of implementing such technologies for clinical practice and everyday life.

 The use of association data by some companies or tests (rather than direct genomic data) is not dwelt upon in the section on DTC genetic testing although this is highlighted in the referenced literature. The distinction is one which is not always clear to the public and relates directly to the question posed at the end of this section about the lay person's genetic/genomic literacy and how well-equipped they are to evaluate such tests themselves.

 Some consumers also use DTC testing from an Ancestry point of view and do not initially set out to learn medical genetic information from the test. However, tools which put related users in touch with each other can sometimes lead to disclosures of more medically-related information, such as recommendations about screening for inherited conditions.

 The authors rightly highlight tensions between genomic data donated by patients to research endeavours on an altruistic basis and the possibility of a resultant financial benefit to organisations who provide capital, resources or facilities to support such endeavours. This is juxtaposed with the concomitant lack of value in being unable to share data for comparison and interpretation.

 Finally, they draw attention to the great need for further research into the impact of these technologies on society as a whole - from a variety of perspectives - particularly focusing on the experiences of end-users, be they patients, consumers or citizens. Understanding the needs and concerns of these stakeholders is vital to successful delivery of services in the future.

I have read this submission. I believe that I have an appropriate level of expertise to confirm that it is of an acceptable scientific standard.
